# Effects of age and diet forms on growth-development patterns, serum metabolism indicators, and parameters of body fat deposition in Cherry Valley ducks

**DOI:** 10.5713/ab.21.0096

**Published:** 2021-06-24

**Authors:** Gang Lv, Qiufeng Zeng, Xuemei Ding, Shiping Bai, Keying Zhang

**Affiliations:** 1 Institute of Livestock and Poultry, Tongwei Co., Ltd., Chengdu, Sichuan-610041, China; 2 Institute of Animal Nutrition, Sichuan Agricultural University, Chengdu, Sichuan-611130, China

**Keywords:** Age, Cherry Valley Ducks, Development, Diet Forms, Fat Deposition, Growth

## Abstract

**Objective:**

This study was conducted to investigate the effects of age and diet forms on growth-development patterns, serum metabolism indicators, and parameters of body fat deposition in Cherry Valley ducks.

**Methods:**

According to the hatching age and initial weight, a total of 150 1-day-old male SM3 Cherry Valley ducks were randomly assigned to two diet forms (pellet vs powder form). Each treatment had with 5 replicates per treatment and 15 meat ducks per replicate. The study lasted 42 d, which was divided into two periods (1 to 21 vs 22 to 42 d).

**Results:**

Our results showed that compared with powder group, ducks in pellet group had greater growth performance during different period (p<0.05). The inflection point was 24 d and was not numerically affected by diet forms. Increasing age (42 vs 21 d) significantly increased the weight of body fat and hepatic fat metabolism related enzyme activities in ducks (p<0.05), meanwhile, increasing age (42 vs 21 d) improved serum metabolism indicators and decreased mRNA expression levels of fat metabolism-related genes in liver (p<0.05). Ducks fed different diets (pellet vs powder form) increased growth performance as well as the weight of body fat and improved serum metabolism indicators (p<0.05). In addition, interactions were found between age and diet forms on the levels of serum metabolism indicators in ducks (p<0.05).

**Conclusion:**

In conclusion, powder feed reduced growth performance of ducks, and the day of inflection point was 24 days old. Ducks with higher age or fed with pellet diet showed higher fat deposition. The effect of age and feed forms on body fat deposition might result from changes in the contents of serum metabolism indicators, key enzyme activity of lipid production, and hepatic gene expressions.

## INTRODUCTION

With the deeper research of animal nutrition science, production performance and feed utilization rate of animals have been greatly improved. However, new problems (excessive body fat deposition, decline in meat quality etc.) subsequently appear, which requires researchers to strengthen the relevant basic research. Over the past two decades, improving the status of body fat deposition in animals has become a research focus, since excessive body fat deposition could not only reduce meat quality and feed utilization rate of animals but threaten animal health, and resulting in serious human health damage and subsequently critical economic losses to society [1,2].

Fat, an essential component of animal muscle, plays an important role in meat quality. The status of animal fat deposition is affected by species, sex, age, and nutrition levels. Moreover, fat deposition in animals can influence feed cost, meat quality, and human health. Compared with chickens, ducks have a stronger ability to transform energy into fat, which is deposited as body fat, especially more subcutaneous fat (SF) to prevent body heat losses in cold water [3]. During the growth process of ducks, weight of carcass muscle and fat increases with age, and fat proportionately grows more than muscle, in addition, ducks show the highest percentage of SF and lowest percentage of breast meat in poultry [4]. Furthermore, modern animal breeding practices have proven to exert beneficial influences on the carcass slaughter rate of ducks, including increases in lean meat and breast meat, although it remains much lower than chicken. It is thus clear that the body fat deposition ability of ducks is far greater than that of chickens [5]. Since ducks have a strong ability to deposit fat, the general approaches commonly adopted to regulate fat metabolism of ducks by nutritionists is to employ feed restriction, adjust the ratio of energy to protein or amino acids, and supply certain additives (soybean phospholipid, betaine, carnitine, conjugated linoleic acid, organic chromium etc.), to reduce abdominal fat (AF) and SF deposition and increase fat deposition in muscle [6]. However, there are few studies on how to appropriately regulate body fat deposition of ducks, therefore, to control body fat in ducks, it is necessary to use growth models to investigate the growth-development patterns, body fat deposition and related mechanisms.

Fat metabolism is a complex dynamically balanced process, and fat deposition is the result of its comprehensive effects. Previous reports have revealed that the fat deposition of ducks increased with the increasing of age [7]. Powder feed is rarely used in meat duck production, which would similarly reduce feed intake and resulting in lower body weight (BW) of meat ducks. In addition, researchers always employ different feeding models (such as starvation, feed restriction, and overfeeding) to investigate animal growth and development status. It was reported that in contrast to feed *ad libitum*, appropriate feed restriction could decrease body fat deposition, which improved meat quality and animal health [8]. Although regulating body fat deposition has received extensive attention in other poultry, interestingly there is no relevant research evaluating the effects of age and low feed intake caused by different diet forms on growth-development patterns, serum metabolism indicators, parameters of body fat deposition, and related mechanisms in ducks.

The objectives of this research were to investigate the effects of age and low feed intake caused by different diet forms on growth-development patterns and fat deposition status in a duck model. The results of this study may contribute to our further understanding of the underlying mechanism of body fat deposition and help to clarify the appropriate application of feed restriction in duck production.

## MATERIALS AND METHODS

### Animal care

All animal procedures associated with this study were approved by the Animal Care and Use committee, Sichuan Agricultural University (Ethic Approval Code: SICAUAC 202003-1; Chengdu, China).

### Experimental design and animal management

A total of 150 one-day-old Cherry Valley male ducks (commercially purchased from Sichuan Guiliu Poultry Co., Ltd., Chengdu, China) was used in a 42-d experiment. At the beginning of the experiment, ducks were randomly assigned to 2 treatments with 5 replicate pens based on their initial BW. The 2 treatment groups were fed a 2-stage basal diet with pellet or powder form.

All ducks were reared in cages (1.2×1.0×0.6 m) in a temperature-controlled room with a 24 h constant light schedule and had free access to water and feed throughout the experimental period, and temperature of the room during 1 to 3 d, 4 to 7 d, 8 to 42 d was maintained at 31°C±1°C, 29°C±1°C, and 27°C±1°C, respectively, and relative humidity of the room was controlled at 60% to 70%. This trial was divided into 2 phases, with a specific diet for each phase: phase 1 (from 1 to 21 d) and phase 2 (from 22 to 42 d), and the 2-stage basal diet (Table 1) was formulated to meet or exceed the nutrient requirements recommended by the National Research Council [9]. After impurity removal, smashing and mixing, the pellet diet was prepared by granulating at 80°C using an automatic granulator device (MUZL-1200, Jiangsu Muyang Group Co., Ltd., Yangzhou, China), the heated feed after granulation was then be cut into pellets with the same particle size by the cutter, and the particle diameter of the pellets on phase 1 and phase 2 was 2.0 mm and 3.0 mm, respectively. Furthermore, the powder diet for each phase was prepared by smashing the finished pellet diet for a phase with a same 3.0-mm screen, thus, diet composition for pellet and powder form was the same.

### Sampling

The ducks were weighed and recorded in the morning on d 0, 8, 15, 22, 29, 36, and 43. Feed intake was recorded daily for each replicate. The data collected were used to calculate the average daily gain (ADG), average daily feed intake (ADFI), accumulative feed intake (AFI), average daily metabolizable energy intake (ADMEI), accumulative metabolizable energy intake (AMEI), and feed conversion ratio (FCR). Mortality was recorded to correct ADFI and FCR.

On d 22 and 43, prior to the morning feeding and following overnight fasting, 2 ducks with the average BW in each pen were weighted, chosen, and bled. Blood samples were collected from the precaval vein into nonheparinized vacuum tubes. Briefly, after centrifugation (3,500× g for 10 min at 4°C), serum samples were collected and stored at −20°C for serum parameters analysis. After bleeding, the same ducks were sacrificed by cervical dislocation, the carcasses were opened and the AF pads, SF pads were removed and weighed according to the previous study by Zhang et al [10]. The percentage of the weight of AF and SF pads were calculated as follows: weight of pads/BW ×100. About 2 g SF and AF samples were respectively collected to determine lipoprotein lipase (LPL) activity. At the same time, approximate 4 g liver samples were collected from the left liver and frozen quickly in liquid nitrogen and then stored at −80°C for RNA extraction and activities of fat deposition-related enzyme.

### Serum physiochemical parameters

Serum glucose (GLU), total cholesterol (TC), triglyceride (TG), high-density lipoprotein (HDL), low-density lipoprotein (LDL), and phospholipid (PL) were measured using commercially available kits (Nanjing Jiancheng Bioengineering Institute, Nanjing, China) and an automatic biochemical instrument (Biochemical Analytical Instrument, Beckman CX4; Beckman Coulter Inc., Brea, CA, USA), and the content of very low density lipoprotein (VLDL) was calculated based on the levels of TC, HDL and LDL (VLDL = TC−HDL−LDL). Furthermore, Serum insulin, glucagon, and leptin levels were assayed using commercially available ELISA kits (Beijing Chenglin Biotechnology Co., Ltd., Beijing, China) and an automatic biochemical instrument (Biochemical Analytical Instrument, Beckman CX4; Beckman Coulter Inc., USA). All measurements were conducted in triplicate at minimum according to the manufacturer’s instructions.

### Activities of lipoprotein lipase in fat tissues and lipogenic enzymes in liver

Approximately 1 g of frozen SF, AF, and liver samples were respectively weighed and homogenized with nine times the volume (wt/vol) of pre-cooled physiological saline. The mixture was centrifuged at 4,000×g for 10 min at 4°C, to collect the supernatant solution. The supernatant protein concentration was assayed using a protein quantification kit (Nanjing Jiancheng Bioengineering Institute, China) as the protein standard. Subsequently, in the supernatant solution, activities of LPL in SF and AF as well as fatty acid synthetase (FAS), malic enzyme (MLE), and glucose-6-phosphate dehydrogenase (G6PDH) in liver were analyzed using commercial kits (Nanjing Jiancheng Bioengineering Institute, China) combined with a UV-VIS Spectrophotometer (UV1100; MAPADA, Shanghai, China) according to the manufacturer’s instructions. All measurements were conducted in triplicate at minimum according to the manufacturer’s instructions.

### Total RNA extraction and real-time quantitative polymerase chain reaction

Liver samples (approximately 0.1 g) were homogenized in 1 mL RNAiso Plus reagent (TaKaRa, Dalian, China), and total RNA was extracted according to the manufacturer’s protocols. The concentration and quality of total RNA were assessed using a spectrophotometer (Beckman Coulter DU 800; Beckman Coulter Inc., USA), determining an optical density (OD)_260_: (OD)_280_ ratio ranging from 1.8 to 2.0 in all RNase-free water-treated RNA samples. Meanwhile, the integrity of RNA was checked by formaldehyde gel electrophoresis, and the 28S: 18S ribosomal RNA band was determined as ≥1.8, then the synthesis of the first strand of cDNA of each sample was obtained by reverse transcription using a PrimeScript reverse transcription reagent kit (TaKaRa, China) following the manufacturer’s instructions.

Specific primers for the acetyl-coA carboxylase (ACC), FAS, MLE, G6PDH, sterol regulatory element-binding protein 1 (SREBP1), carbohydrate response element binding protein (ChREBP), and peroxisome proliferator-activated receptor (PPAR-α) were designed and purchased from Invitrogen (Shanghai, China), which are listed in Table 2. The real-time polymerase chain reaction (PCR) reactions were performed on CFX96 Real-Time PCR Detection System (Bio-Rad Laboratories, Inc., Hercules, CA, USA), using SYBR Green PCR reagents (TaKaRa, China). A total volume of 10 μL PCR reaction system was comprised of 5 μL SYBR Green, 0.5 μL forward primer, 0.5 μL reverse primer, 1 μL cDNA and 3 μL nuclease-free H_2_O. The real-time PCR reactions were performed using the following cycle program: a precycling stage at 95°C for 30 s, and 40 cycles of denaturization at 95°C for 10 s and annealing at annealing temperature for 25 s with a final extension at 72°C for 5 min.

A melting curve analysis was generated following each real-time quantitative PCR assay to check and verify the specificity and purity of all PCR products. The reference gene transcript (β-actin) was chosen as the reference gene to normalize cDNA loading. For calculation of the amplification efficiencies, a 10-fold serial dilution was used to generate standard curves for both targeted and reference genes, quantifying six concentrations. After verification that the primers amplified with an efficiency of approximately 100%, and the results were analyzed using the 2^−ΔΔCt^ method [11]. Analysis of each standard and sample were run in triplicate simultaneously on the same PCR plate, and the average of each triplicate value expressed as numbers of copies was used for subsequent statistical analysis.

### Statistical analysis

Growth performance data were analysed with independent T-test using the statistical program of SAS 9.0 software (SAS software; SAS Institute, Inc., Raleigh, NC, USA) with pen as the experimental unit (n = 5). Quadratic regressions with curve estimation procedure of SAS 9.0 software (SAS, USA) were employed to investigate the relationship between age and growth performance (including ADG, ADFI, AFI, ADMEI, and AMEI). The growth curves were fitted as nonlinearity regression equation using typical Von Bertalanffy S curve model. Residual sum of squares (RSS) and coefficient of determination (R^2^) were used to evaluate the goodness of fittest. The curve model was as follow: W_t_ = W_A_×[1−Bexp(−Kt)]^3^ (W_t_, weight at t time; W_A_, ultimate weight; B, regulation parameter; K, exponential growth ratio; (ln3B)/K, day of inflection point; 8W_A_/27, BW of 8W_A_/27). All other data were also analyzed by two-way analysis of variance using the Generalized Linear Models procedure of SAS 9.0 software (SAS, USA) with average data of 2 sampled ducks per pen as the experimental unit (n = 5). The statistical model included the main effects of age, diet form, and their interaction. The results were presented as mean and standard error of means. Statistical differences among treatment were determined by Duncan’s multiple-range test. For significance determination, the α-level was set as 0.05. Probability values less than 0.05 were considered significant, whereas probability values less than 0.10 was considered a tendency.

## RESULTS

### Growth performance

No death occurred in neither pellet nor powder fed ducks throughout the trial. As shown in Table 3, compared with powder group, ducks in pellet group had greater ADFI during 1, 2, 3, 6, 1 to 3, and 1 to 6 weeks (p<0.05). In addition, ducks fed pellet diets exhibited higher ADG during 1, 2, 3, 6, 1 to 3, 4 to 6, and 1 to 6 weeks than those fed the powder diet (p<0.05). Thus, pellet diet significantly decreased FCR of ducks during 1, 2, 3, 4, 6, 1 to 3, 4 to 6, and 1 to 6 weeks compared with powder diet.

### Feed intake curves

**Figure 1:**
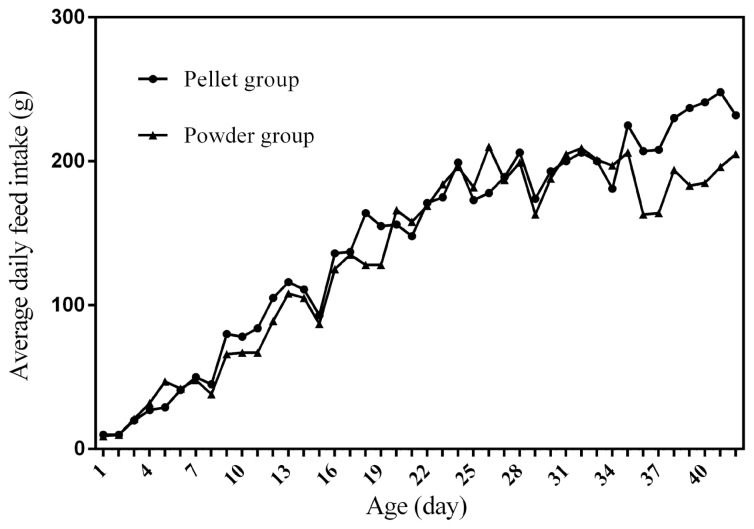
The curve of average daily feed intake in ducks fed different form diets. ADFI, average daily feed intake; t, age; R^2^, coefficient of determination. The equation for predicting ADFI of ducks fed the pellet diet: ADFI (g) = −0.1059t^2^+10.211t−10.123, R^2^ = 0.9699. The equation for predicting ADFI of ducks fed the powder diet: ADFI (g) = −0.1633t^2^+11.927t−21.624, R^2^ = 0.9430.

**Figure 2:**
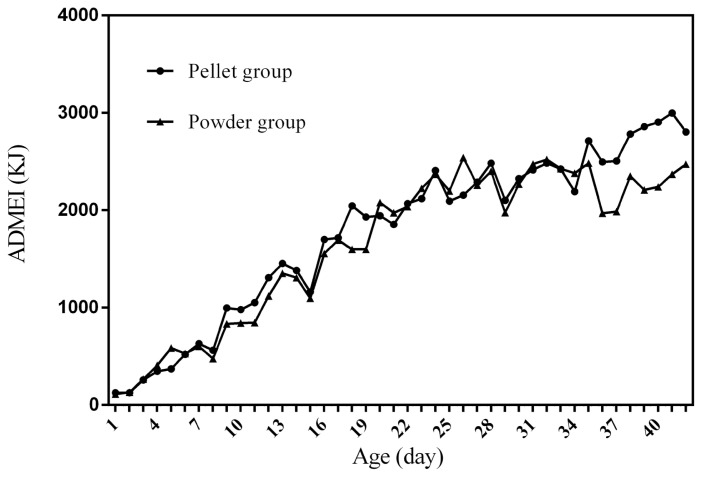
The curve of average daily metabolizable energy intake in ducks fed different form diets. ADMEI, average daily metabolizable energy intake; t, age; R^2^, coefficient of determination. The equation for predicting ADMEI of ducks fed the pellet diet: ADMEI (KJ) = −1.3494t^2^+125.4t−110.5, R^2^ = 0.9674. The equation for predicting ADMEI of ducks fed the powder diet: ADMEI (KJ) = −2.0347t^2^+145.81t−248.99, R^2^ = 0.9446.

**Figure 3:**
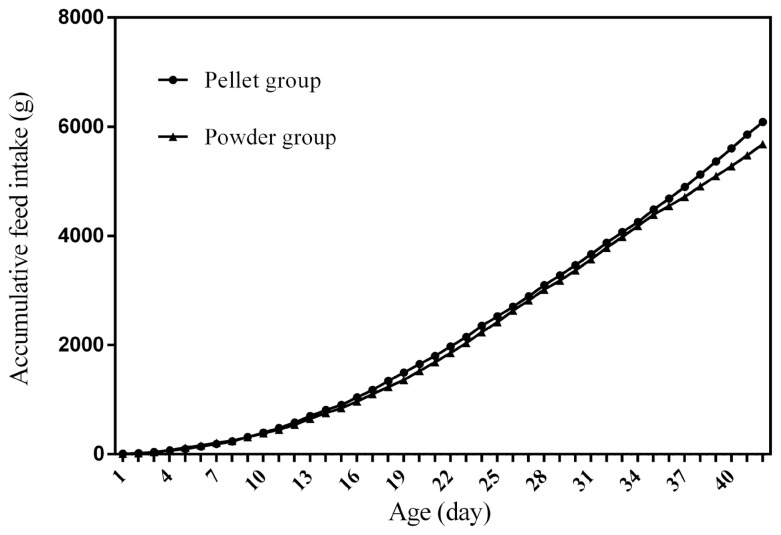
The curve of accumulative feed intake in ducks fed different form diets. AFI, accumulative feed intake; t, age; R^2^, coefficient of determination. The equation for predicting AFI of ducks fed the pellet diet: AFI (g) = 2.7117t^2^+37.374t−170.76, R^2^ = 0.9988. The equation for predicting AFI of ducks fed the powder diet: ADFI (g) = 2.5306t^2^+38.528t−181.21, R^2^ = 0.9969.

**Figure 4:**
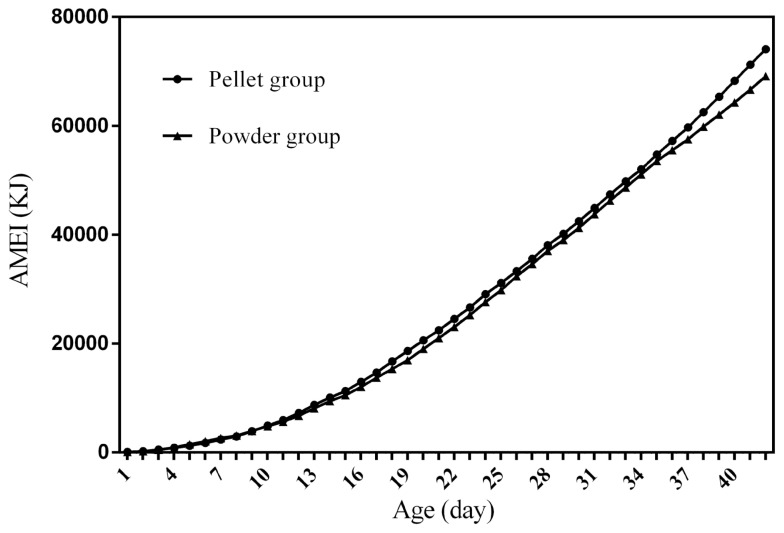
The curve of accumulative metabolizable energy intake in ducks fed different form diets. AMEI, accumulative metabolizable energy intake; t, age; R^2^, coefficient of determination. The equation for predicting AMEI of ducks fed the pellet diet: AMEI (KJ) = 31.919t^2^+503.99t−2260.3, R^2^ = 0.9987. The equation for predicting AMEI of ducks fed the powder diet: AMEI (KJ) = 29.8t^2^+513.69t−2365, R^2^ = 0.9969.

Figure 1 to 4 represent the curves of ADFI, ADMEI, AFI, and AMEI. The ADFI and ADMEI which showed strong regular changes, and could be well fitted as quadratic multinomial equation of age (p<0.05 and R^2^>0.94). However, FI of meat ducks did not show a monotonically increasing trend, but showed a zigzag-increasing trend. Additionally, the variation degree of FI in ducks fed pellet diet was lower than that of ducks fed powder diet, and whose fitted equation showed a higher coefficient of determination. Fitting degree of quadratic curve regressions in AFI and AMEI were higher than those in ADFI and ADMEI (accumulative intake, R^2^>0.99 vs daily intake, R^2^>0.94), which indicated that the regularity in curves of AFI and AMEI was stronger and with a higher reliability than that of ADFI and ADMEI. Briefly, regardless of the feed intake or energy intake, the intake of ducks fed pellet diet was higher than those fed powder diet.

### Growth curve fitted as Von Bertalanffy model

As shown in Table 4, Von Bertalanffy model perfectly fitted the growth curve of ducks. Higher fitting degree was observed in ducks fed pellet diet than those of ducks fed powder diet (pellet form, R^2^ = 0.9996 vs powder form, R^2^ = 0.9980), in addtion, they had large variance between predictive value and practical value on 1 d, but lower variance was observed after 7 d, which indicated that the prediction value had strong predictability after 7 d. Furthermore, the final weight of ducks was numerically effected by feed form (pellet form, W_A_ = 5,068.42 g vs powder form, W_A_ = 3,847.44 g), however, the day of inflection point was not numerically effected by diet form (pellet form, t = 24.78 d vs powder form, t = 23.74 d), which indicated that the growth and development of ducks was inhibitted by powder diet compared with pellet diet. Accordingly, the growth velocity of ducks increased gradually before 24 d, after which the growth velocity of ducks decreased.

### Status of body weight deposition

The status of BW deposition is given in Table 5. Increasing age (42 vs 21 d) significantly increased the weight of BW, AF, SF, and the ratio of AF/BW in ducks (p<0.05). Ducks fed different diets (pellet vs powder form) increased the weight of BW, AF, SF as well as the ratio of AF/BW, and decreased the ratio of SF/BW (p<0.05). In addition, interactions were found between age and diet form on the weight of BW in ducks (p<0.05).

### Serum physiochemical parameters

As shown in Table 6. Increasing age (42 vs 21 d) had higher serum levels of GLU as well as leptin, and lower contents of TC, HDL, LDL, VLDL, and PL (p<0.05). Ducks fed different diets (pellet vs powder form) deceased the levels of GLU, TC, HDL as well as LDL in serum, and increased the serum content of leptin (p<0.05). In addition, interactions were found between age and diet form on the serum levels of TC, TG, HDL, and glucagon of ducks (p<0.05).

### Activities of fat metabolism related enzymes in fat and liver tissues

The activities of fat metabolism related enzymes data are given in Table 7. The LPL activity in abdominal fat or SF was not effected by age, diet form, and their interactions (p>0.10). However, in liver tissue, there were higher FAS and MLE activities in 42-d-old ducks than those of 21-d-old ducks (p< 0.05).

### Gene expression of fat metabolism-related genes

Table 8 represents the differences in mRNA level of fat mebolism-related genes between the 4 groups. Increasing age (42 vs 21 d) had lower mRNA expression levels of ACC, MLE, and PPAR-α in liver of duck (p<0.05). Ducks fed different diets (pellet vs powder form) had no significant differences. However, there was no interaction between age and diet form on mRNA level of fat metabolism-related genes in liver of ducks (p>0.10).

## DISCUSSION

Feed intake, as a critical element for feed to play nutritional roles, has been a necessary indicator for nutritionists to determine the appropriate dietary nutrient concentration for decades. Furthermore, the primary condition for predicting the dynamic nutritional need of animals is to establish a prediction model of animals’ FI (expressed as ingestion value of feed weight or energy). In the primary study, we investigated the scatter diagram of ducks’ FI, then employed quadratic polynomial to fit ADFI, AFI, ADMEI, and AMEI of ducks, which obtained great fitting effects, and the fitting degrees for accumulate intake were higher than those of daily intake. Similar findings were observed on chicks by other researchers, and they reported that the linear function (age as independent variable) could accurately predict the daily feed intake of 0 to 21 d chicks, while the accumulated intake of 0 to 21 d chicks could be better predicated by quadratic polynomial than that of linear function [12]. However, in the primary study, a higher fitting degree was observed in ducks fed pellet diet than those fed powder diet (pellet form, R^2^ = 0.9996 vs powder form, R^2^ = 0.9980), which indicated that the FI of ducks fed powder diet might receive a greater challenge, resulting in poor regularity. Previous studies indicated that pellet diet could improve FI and subsequent performance in pigs [13], which was consistent with our results in the primary study that ducks in pellet group had greater growth performance than those in powder group. Animal feed intake is generally affected by many factors (species, sex, health status, environment, dietary nutrition concentration, feed form etc.), however, when these factors are relatively stable, FI is mainly regulated by BW and age. NRC (2012) presented regression equation to predict FI of sucking piglets based on age [14], similarly, in the present study, the FI of ducks was predicted with age increasing, which had accurate reliability and could be applied as references in duck production.

The growth and development of animals show s-shaped curve changes with age increasing, thus, investigating and establishing growth curve models of animals plays a key role in the adoption of phased feeding and timely slaughter in animal production, which similarly is a prerequisite for the accurate prediction of dynamic nutrition requirement of animals. The combination of animal growth curve model and FI data could be employed to predict the weight growth rate, feed consumption, and feed conversion rate of livestock and poultry in every growing period, which could help researchers to effectively determine the influence of external factors such as nutrition and environment on animal growth. The s-shaped growth curve of poultry consists of 2 periods: i) a period of accelerated growth from hatching to the emergence of inflection point, and poultry at the inflection point have the maximum growth rate; ii) after period 1, the growth rate begin to decline, meanwhile, a limit value of growth (mature BW) would appear with age increasing. Recent years, researchers have studied the growth curve models of chickens from different breeds, strains, and feeding conditions, however, relatively few studies of growth curve models have been conducted on meat ducks [15]. In the present study, it was found that the Von Bertalanffy model could approximately fit the growth curve of ducks. Furthermore, the age of inflection points in ducks fed the 2 different diet forms was close (pellet form, 24.783 d vs powder form, 23.738 d), which indicated that the powder form diet did not change the shape of growth curve, but reduced the mature BW of meat duck, thus it is negative to increase the mature BW of ducks by prolonging the feeding period. As for growth performance, the maximum ADG of each week (pellet form, 89.799 g/d vs powder form, 83.827 g/d) in ducks was obtained in the 4th week (22 to 28 d), which further proves the credibility of calculated inflection point age by growth curve. In addition, after the inflection point age, the ADG of ducks was reduced with increasing ADFI, resulting in increasing FCR, which was proven by the animal trial (Table 3) that ADG of ducks fed pellet and powder diets during 29 to 35 d was reduced by 25.54% and 27.96% respectively than those during 22 to 28 d, which was a bigger decline than in any other consecutive week. Accordingly, 4th week (22 to 28 d) might be the most appropriate period for ducks to be slaughtered. Meanwhile, the primary experiment found that the ADG, ADFI, and FCR in ducks fed powder diet was reduced by 19.5%, 6.8%, and 16.5% respectively compared to those ducks fed pellet diet, which was in line with another study on ducks [16], indicating that the powder diet was not suitable for the ingestion habits of ducks and was not conducive to promotion in the meat duck industry.

Meat ducks can better convert surplus energy into fat deposited in subcutaneous and internal organs compared with broilers, which could prevent energy lose. In the primary study, the result showed that the increasing age (42 vs 21 d) significantly increased the weight of AF, SF, and the ratio of AF/BW in ducks, which indicated that the body fat deposition status of ducks was significantly improved with age increasing, these results were consistent with previous study [16]. However, the primary study indicated that the increasing age (42 vs 21 d) numerically decreased the ratio of SF/BW in ducks, Zhang et al [17] showed that the proportion of SF/carcass weight in Gaoyou ducks reached the highest value at 3 weeks old and the SF changed little during the whole trial weeks. Conversely, Bochno et al [16] showed that the proportion of SF/carcass weight in Peking ducks changed little during 2 to 13 weeks while SF changed greatly with the increase of age, the reason for which might consist of the differences between breeds and calculation methods. In addition, it is worth mentioning that the skin and SF are generally used as indicators to evaluate status of carcass fat deposition. However, in fact, there are differences in the development of skin and SF in animals [16,17], consequently, it might be inaccurate to employ sebum rate as a general indicator of body fat deposition. Furthermore, in our study, ducks fed different diets (pellet vs powder form) increased the weight of BW, AF, and SF as well as the ratio of AF/BW, and decreased the ratio of SF/BW, indicating that the powder diet reduced the body fat deposition indicators of ducks compared with those of ducks fed with pellet diet, which may be related to the lower feed intake and subsequent reduced energy intake caused by powder diet. Accordingly, both age and feed form had effects on status of body fat deposition in ducks, and the effects on AF deposition were greater than those on SF deposition.

Fatty acid synthesis of poultry mainly occurs in liver, and the accumulated fat mainly comes from de novo synthesis in the liver because of the limited addition of exogenous fat in the diet. However, lipids de novo synthesized in the liver of poultry must be transported to extrahepatic tissues by lipoprotein, there to be used by fat and muscle tissues, indicating that the type and content of lipoprotein in the serum might correlate to body fat deposition. In this study, it was found that ducks with increasing age (42 vs 21 d) had lower serum contents of TC, HDL, LDL, and VLDL, this was in line with research of a previous study [18]. Kocan and Pitts [19] reported that ducks had higher serum levels of GLU with increasing age, which agreed with our results. The relatively high content of serum GLU might promote fatty acid synthesis in liver through 2 ways: i) directly stimulating the gene expression of fatty acid synthase in liver or acting on insulin to play a role, therefore, this effect of serum GLU may eventually lead to an increase in serum TG content, which was coincided with our results. ii) There is a strong correlation between body fat deposition and serum leptin content, and insulin can stimulate the secretion of leptin [20]. The results in the primary study showed that the concentration of leptin in serum of meat ducks was increased with age, which was consistent with the trend of increasing AF and SF deposition of ducks. Likewise, the specific mechanism causing the older ducks to have lower serum TC content remains to be further studied. However, in this study, it seemed that there was a negative correlation between serum TG concentration and body fat deposition of meat ducks, as the powder diet reduced the body fat deposition but increased serum TG content indictors. Similarly, the body fat deposition status of 42-d-old ducks was generally higher than that of 21-d-old ducks, however, the serum cholesterol metabolism indexes of 42-d-old ducks showed lower levels than those of 21-d-old ducks. The LPL, a proteolytic enzyme, synthesized by adipocytes, skeletal muscle cells, mammary gland cells, and other parenchymal cells, is widely distributed in different tissues and has high content in adipose tissue and skeletal muscle. The LPL is mainly synthesized and secreted in adipose tissue and skeletal muscle, which reaches the capillary wall and subsequently has physiological activities. The LPL can hydrolyze TGs carried by chylomicrons and VLDL in the blood into glycerol and fatty acids. The separated free fatty acids enter the adipose tissue, are re-esterified, and are preserved in TGs form. In our study, the activity of LPL in AF of 42-d-old ducks was numerically higher than that of 21-d-old ducks, which was inconsistent with the study on domestic ducks [21]. However, in line with our study, Sato et al reported that short-term feeding restriction and subsequently returning to normal feeding after restriction had no effect on genes and protein expression of LPL in AF [22]. In addition, the primary study showed that LPL activity in AF of ducks was higher than that in SF of ducks, suggesting that AF deposition rate may be higher than SF deposition rate in ducks.

The FAS, one of the key enzymes for fatty acid synthesis in liver, can catalyze the synthesis of malonyl coenzyme A from acetyl coenzyme A, thus synthesizing TG, which however requires the presence of acetyl CoA carboxylase. Meanwhile, reduced nicotinamide adenine dinucleotide phosphate (NAPDH) plays an irreplaceable role in this process, as NADPH is an important coenzyme in fatty acid synthesis and the extension of carbon chains in animals and is the hydrogen supplier in fatty acid reductive synthesis. The main sources of NADPH in animal tissues are as follows: i) in the pentose phosphate pathway, the reaction catalyzed by G6PDH and 6PGDH transfers NADP to NADPH; ii) in the malic pyruvate cycle, MLE catalyzes malic acid to pyruvate, this cycle could produce NADPH. Therefore, FAS, G6DPH, and MLE become the key enzymes and rate limiting enzymes of fatty acid synthesis in liver. In the primary study, increasing age (42 vs 21 d) had higher hepatic activity of FAS, indicating that the ability of liver to synthesize fatty acids may enhance with the increase of age, which is consistent with the result of Bazin and Lavau [23] who reported that FAS activities in liver of 7, 9, 14, and 17 d-old rats showed significant differences. Baeza et al [24] found that the hepatic FAS activity of mule ducks increased rapidly with age during growing, and there was a significantly positive correlation between them, and was consistent with the results of the primary study. In addition, increasing age (42 vs 21 d) also tended to increase the activity of G6DPH in liver, which was consistent with the study on mice [25]. In the primary study, the results suggested that the activity of G6DPH in liver was numerically higher in the pellet group than that in powder group and might correlate to the higher FI in pellet group, this was consistent with the results on mice of Amir-Ahmady and Salati [26]. Meanwhile, we also found that increasing age (42 vs 21 d) significantly increased hepatic MLE activity. High carbohydrate content is one of the factors regulating MLE transcription in liver tissue, the concentration of nutrients ingested by birds is positively correlated with the expression of MLE in liver. In the primary experiment the GLU concentration in serum of ducks was significantly increased with increasing age, which might lead to a significant increase in malate dehydrogenase activity in liver tissue. However, the key enzyme gene expressions of lipogenesis in this experiment did not show the same regulating rule with enzyme activity, suggesting that the difference of enzyme activity might not be caused by gene expression difference, as there were still other pathways (protein expression and modification, etc.) to regulate enzyme activity.

The activation of lipase gene transcription requires the interaction of insulin and GLU. ChREBP is a transcription factor in the GLU signaling pathway, and plays an important role in the process of fat metabolism and cooperates with SREBP to regulate the expression of fatty acid synthase gene. In the primary study, it could be shown that the expression of ChREBP significantly decreased with the increase of age, which was different from the research in previous study [27], the possible reason was that ChREBP not only regulated glycolysis and fat production pathways, but also affected the genes involved in β-oxidation as well as energy storage. In addition, it was also possible that the effects of age and feed forms on ChREBP expression were more likely to play a role in post-transcriptional control stage [28]. The expression of *ChREBP* gene in the liver is affected by many factors, such as GLU, insulin, liver X receptor, etc., which upregulates ChREBP, while PUFA and PPAR α inhibit ChREBP’ regulation of GLU response gene. An important pathway of insulin regulating gene expression is realized through SERBPs. SREBP-1 is the intermediate of insulin regulating the expression as well as activity of glucokinase gene and the long-term regulation of lipogenic gene expression, while the abundance of SREBP-1 expression depends on the concentration of insulin *in vivo* [29]. In the results of our experiment, it could be seen that age or feed form have no significant effect on the concentration of serum insulin and the expression of SREBP-1. However, with the increase of age, the expression of SREBP-1 tended to decrease, suggesting that the regulation of SREBP-1 expression by insulin might be in post-transcriptional control stage. Meanwhile, PPARs play an important role in regulating animal fat metabolism, especially fatty acid oxidation and adipocyte differentiation. PPARα is mainly expressed in the liver, heart, muscle, and kidney, regulating the decomposition and metabolism of fat. The primary results showed that increasing age (42 vs 21 d) had higher mRNA expression level of PPAR-α in liver of ducks, and ducks fed different diets (pellet vs powder form) tended to decrease mRNA expression levels of PPAR-α in liver, those differential expressions indicated that fatty acid oxidation rate of meat ducks at 42 d might be lower than that of ducks at 21 d, and the fatty acid oxidation rate in pellet group might also be lower than that in powder group. The decrease of fatty acid oxidation rate in liver can result in the increase of fat deposition, therefore, in the primary experiment, the effects of age and feed form on fat tissue deposition of ducks might correlate to the expression of *PPAR-α* gene in liver.

In conclusion, powder feed reduced growth performance of ducks. Von Bertalanffy model perfectly fitted the growth curve of ducks, the day of inflection point was not numerically effected by diet forms and and the inflection point was 24 days old. Ducks with higher age or fed with pellet diet showed greater fat deposition. The effect of age and feed form on body fat deposition might result from changes in the contents of serum GLU, TG, insulin, leptin, key enzyme activity of lipid production and hepatic SREBP-1, ChREBP, and PPAR-α gene expressions.

## Figures and Tables

**Table 1:** Diet compositions (%, as fed basis)

Item	Phase 1	Phase 2
Maize	58.37	62.87
Soybean meal	26.30	13.00
Rapeseed meal	5.00	8.00
Wheat bran	5.00	12.00
Soybean oil	2.00	1.00
Calcium carbonate	0.70	0.80
Dicalcium phosphate	1.10	0.80
Sodium bicarbonate	0.30	0.30
Choline chloride	0.20	0.20
L-Lysine HCl	0.10	0.15
DL-Methionine	0.20	0.15
NaCl	0.30	0.30
Preservative^1)^	0.10	0.10
Vitamin premix^2)^	0.03	0.03
Mineral premix^3)^	0.30	0.30
Total	100	100
Calculated nutrients^4)^ (%)		
ME (kJ/kg)	12,049	11,629
CP	19.10	15.85
Ca	0.73	0.66
TP	0.67	0.66
AP	0.41	0.34
Apparent ileal digestible amino acid (%)		
SID-Lys	0.95	0.75
SID-Met	0.48	0.38
SID-Met+Cys	0.79	0.66
SID-Thr	0.72	0.58
SID-Trp	0.22	0.17

ME, metabolizable energy; CP, crude protein; TP, total phosphorous; AP, available phosphorous.

1) Provided per kilogram of preservative: sodium diacetate, 1,000 mg.

2) Provided per kilogram of diet: vitamin A, 5,000 IU; vitamin D_3_, 400 IU; vitamin E, 10 IU; vitamin K_3_, 0.5 mg; vitamin B_1_, 2.0 mg; vitamin B_6_, 2.5 mg; vitamin B_12_, 0.02 mg; nicotinic acid, 55 mg; pantothenic acid, 10 mg; folic acid, 1.0 mg; and biotin, 0.1 mg.

3) Provided per kilogram of diet: 80 mg Fe; 20 mg Cu; 60 mg Zn; 60 mg Mn; 0.2 mg Se; and 0.2 mg I.

4) Values are calculated.

**Table 2:** Sequence of primers

Genes	Primer sequences (5′-3′)	Size (bp)	AT (°C)	Accession number
*ACC*	F: CTGGTGAAGACAATGAGGAGAG	147	60	EF990143
	R: CTGGTGGTAAATGGGAATCAGG			
*FAS*	F: CTCCTCCAGTCTCATGGCTCTA	143	60	AY613443
	R: CAGGACTAAGCATACCCAGCTT			
*MLE*	F: CAGATTGCTTACTCCCTGCTCT	124	62	X66418
	R: CCATCACTACGCCTTCCAAAAC			
*G6PDH*	F: TCTTCAACCCTGAGGAGTC	124	60	AY367543
	R: ACAAAGTGATTTGGCTC			
*SREBP-1*	F: GATGCGTTGGAGTACCTTCAG	168	60	AY613441
	R: GTCACCCTTCAGCCAGTGAAT			
*ChREBP*	F: CTGGAGACCAACAGAGAAATGG	128	58.5	AM883128
	R: AGATGTCCGAGAGGAATGTGTC			
*PPAR-α*	F: AGACACCCTTTCACCAGCATC	143	60.0	EF534215
	R: GTACTCCGTAATGGTAGCCTGAG			
*β-actin*	F: CCCAAAGCCAACAGAGAGAAG	146	60.0	EF667345
	R: GTAACACCATCACCAGAGTCCA			

AT, annealing temperature; F, forward primer; R, reverse primer; *ACC*, acetyl-coA carboxylase; *FAS*, fatty acid synthetase; *MLE*, malic enzyme; *G6PDH*, glucose-6-phosphate dehydrogenase; *SREBP1*, sterol regulatory element-binding protein 1; *ChREBP*, carbohydrate response element binding protein; *PPAR-α*, peroxisome proliferator-activated receptor.

**Table 3:** Effects of age and diet forms on growth performance in ducks

Item	Pellet form	Powder from	SEM	p-value^1)^
1–7 d				
ADFI (g)	27.285	30.251	0.605	0.04
ADG (g)	22.037	16.028	0.401	<0.01
FCR	1.239	1.904	0.037	<0.01
8–14 d				
ADFI (g)	88.752	77.748	1.017	<0.01
ADG (g)	57.733	39.424	0.472	<0.01
FCR	1.537	1.972	0.010	<0.01
15–21 d				
ADFI (g)	141.752	133.037	2.071	0.02
ADG (g)	81.381	65.363	1.626	<0.01
FCR	1.746	2.036	0.032	<0.01
22–28 d				
ADFI (g)	185.099	189.994	2.868	0.23
ADG (g)	89.799	83.827	2.873	0.15
FCR	2.059	2.310	0.060	0.03
29–35 d				
ADFI (g)	197.345	195.944	5.943	0.37
ADG (g)	73.526	69.128	4.470	0.14
FCR	2.765	3.206	0.160	0.10
36–42 d				
ADFI (g)	229.380	184.886	9.999	0.03
ADG (g)	79.159	50.840	10.653	0.03
FCR	2.977	3.592	0.147	0.02
1–21 d				
ADFI (g)	85.930	80.346	1.023	<0.01
ADG (g)	53.717	40.272	0.630	<0.01
FCR	1.600	1.997	0.153	<0.01
22–42 d				
ADFI (g)	203.941	190.274	5.631	0.13
ADG (g)	80.828	67.932	2.886	0.02
FCR	2.548	3.034	0.100	0.01
1–42 d				
ADFI (g)	144.936	135.310	4.675	0.04
ADG (g)	67.272	54.102	1.599	<0.01
FCR	2.129	2.559	0.042	<0.01

SEM, standard error of means; ADFI, average daily feed intake; ADG, average daily gain; FCR, feed conversion ratio.

1) p<0.05 was considered statistically significant.

**Table 4:** The growth curve of ducks fed different form diets fitted as Von Bertalanffy model^1)^

Day (d)	Pellet form			Powder form		
	
	Practical value (g)	Predictive value (g)	Residual error (g)	Practical value (g)	Predictive value (g)	Residual error (g)
1	50.272±0.392	27.293	22.979	49.553±0.432	5.365	44.188
7	204.533±2.68	205.916	−1.383	161.752±3.291	119.293	42.459
14	608.667±3.053	623.571	−14.904	437.724±6.769	460.457	−22.733
21	1,178.333±17.456	1,178.961	−0.628	895.262±9.459	938.930	−43.668
28	1,806.923±21.703	1,776.803	30.120	1,482.051±32.852	1,451.751	30.300
35	2,321.603±41.461	2,351.297	−29.694	1,965.944±45.452	1,932.210	33.734
42	2,875.717±54.285	2,866.649	9.068	2,321.824±80.029	2,348.649	−26.825

BW, body weight; t, age; R^2^, coefficient of determination; W_A_, ultimate weight.

1) The equation fitted as Von Bertalanffy model of growth curve in ducks fed pellet diet: BW (g) = 5,068.423×(1−0.857e^−0.038t^)^3^, R^2^ = 0.9996, inflection t = 24.783 d, infelection BW = 1,501.755 g, W_A_ = 5,068.423 g; The equation fitted as Von Bertalanffy model of growth curve in ducks fed powder diet: BW (g) = 3,847.444×(1−0.927e^−0.043t^)^3^, R^2^ = 0.9980, inflection t = 23.738 d, inflection BW = 1,139.983 g, W_A_ = 3,847.444 g.

**Table 5:** Effects of age and diet forms on status of body weight deposition in ducks

Item	21 d		42 d		SEM	p-value^1)^		
		
	Pellet form	Powder form	Pellet form	Powder form		A	D	A×D
BW (g)	1,184.50^b^	901.00^b^	2,970.56^a^	2,432.78^a^	30.51	<0.01	<0.01	0.01
AF (g)	6.10	2.79	22.57	16.37	1.34	<0.01	<0.01	0.24
SF (g)	202.13	148.16	418.31	347.61	20.51	<0.01	<0.01	0.25
AF/BW	0.51	0.31	0.77	0.67	0.07	<0.01	<0.01	0.61
SF/BW	17.05	16.43	14.21	14.33	1.16	0.66	<0.01	0.51

SEM, standard error of means; BW, body weight; AF, abdominal fat; SF, subcutaneous fat.

1) A, age effect; D, diet form effect; A×D, age×diet form effect.

a,b Means in a row with different letter differ (p<0.05).

**Table 6:** Effects of age and diet forms on serum physiochemical parameters in ducks

Item	21 d		42 d		SEM	p-value^1)^		
		
	Pellet form	Powder from	Pellet form	Powder from		A	D	A×D
GLU (mmol/L)	8.229	8.818	11.030	11.720	0.296	<0.01	<0.01	0.30
TC (mmol/L)	5.143^a^	6.216^a^	3.320^b^	3.326^b^	0.180	<0.01	<0.01	0.01
TG (mmol/L)	1.407^b^	1.283^b^	1.429^b^	1.736^a^	0.096	0.10	0.23	0.03
HDL (mmol/L)	2.389^a^	3.056^a^	1.881^b^	1.770^b^	0.107	<0.01	<0.01	<0.01
LDL (mmol/L)	1.770	2.236	1.127	1.161	0.138	<0.01	<0.01	0.25
VLDL (mmol/L)	0.984	0.924	0.401	0.361	0.039	<0.01	0.26	0.70
PL (mmol/L)	2.869	3.044	2.623	2.616	0.095	<0.01	0.47	0.48
Insulin (μIU/mL)	4.592	4.983	5.283	5.184	0.527	0.08	0.66	0.18
Glucagon (pg/mL)	252.084^b^	276.319^b^	338.050^a^	252.555^b^	20.017	0.22	0.09	<0.01
Leptin (ng/mL)	0.406	0.429	0.791	0.622	0.041	<0.01	<0.01	0.16

SEM, standard error of means; GLU, glucose; TC, total cholesterol; TG, triglyceride; HDL, high-density lipoprotein; LDL, low-density lipoprotein; VLDL, low-density lipoprotein; PL, phospholipid.

1) A, age effect; D, diet form effect; A×D, age×diet form effect.

a,b Means in a row with different letter differ (p<0.05).

**Table 7:** Effects of age and diet forms on activities of fat metabolism related enzymes in fat tissues and liver of ducks

Item	21 d		42 d		SEM	p-value^1)^		
		
	Pellet form	Powder from	Pellet form	Powder from		A	D	A×D
Abdominal fat								
LPL (U/mg)	4.516	4.615	6.541	4.971	0.67	0.11	0.28	0.31
Subcutaneous fat								
LPL (U/mg)	3.981	3.975	3.761	3.816	0.467	0.19	0.68	0.78
Liver								
FAS (nmol/min·mg)	26.134	23.178	43.361	42.781	3.671	<0.01	0.97	0.65
G6PDH (nmol/min·mg)	38.174	33.781	68.267	63.561	5.349	0.08	0.17	0.28
MLE (nmol/min·mg)	110.891	110.897	91.456	145.671	10.571	0.03	0.26	0.56

SEM, standard error of means; LPL, lipoprotein lipase; FAS, fatty acid synthetase; G6PDH, glucose-6-phosphate dehydrogenase; MLE, malic enzyme.

1) A, age effect; D, diet form effect; A×D, age×diet form effect.

**Table 8:** Effects of age and diet forms on mRNA level of fat metabolism-related genes in liver of ducks

Item	21 d		42 d		SEM	p-value^1)^		
		
	Pellet form	Powder from	Pellet form	Powder from		A	D	A×D
*ACC*	1.000	1.024	0.088	0.117	0.123	0.03	0.67	0.23
*FAS*	1.000	2.597	0.347	0.312	0.134	0.28	0.11	0.90
*G6PDH*	1.000	0.865	0.611	0.157	0.434	0.15	0.78	0.61
*MLE*	1.000	1.406	0.364	0.181	0.417	0.02	0.50	0.12
*ChREBP*	1.000	1.285	0.257	0.063	0.009	0.06	0.52	0.66
*SREBP-1*	1.000	1.250	1.500	0.188	0.003	0.06	0.72	0.24
*PPAR-α*	1.000	2.814	0.094	0.183	0.900	0.04	0.07	0.71

SEM, standard error of means; *ACC*, acetyl-coA carboxylase; *FAS*, fatty acid synthetase; *G6PDH*, glucose-6-phosphate dehydrogenase; *MLE*, malic enzyme; *ChREBP*, carbohydrate response element binding protein; *SREBP1*, sterol regulatory element-binding protein 1; *PPAR-α*, peroxisome proliferator-activated receptor.

1) A, age effect; D, diet form effect; A×D, age×diet form effect.
